# Cannabis, tobacco and domestic fumes intake are associated with nasopharyngeal carcinoma in North Africa

**DOI:** 10.1038/sj.bjc.6605281

**Published:** 2009-09-01

**Authors:** B-J Feng, M Khyatti, W Ben-Ayoub, S Dahmoul, M Ayad, F Maachi, W Bedadra, M Abdoun, S Mesli, H Bakkali, M Jalbout, M Hamdi-Cherif, K Boualga, N Bouaouina, L Chouchane, A Benider, F Ben-Ayed, D E Goldgar, M Corbex

**Affiliations:** 1 Genetic Epidemiology Group, International Agency for Research on Cancer, 69372 Lyon, France; 2 Department of Dermatology, University of Utah School of Medicine, Salt Lake City, UT 84132, USA; 3 Laboratoire d'Onco-virologie, Institut Pasteur du Maroc, 20360 Casablanca, Morocco; 4 Association Tunisienne de Lutte Contre le Cancer, 10006 Tunis, Tunisia; 5 Service de Radiothérapie, CHU Farhat Hached, 4000 Sousse, Tunisia; 6 Service de Radiothérapie Oncologique, Centre Anti-cancer de Blida, 09000 Blida, Algeria; 7 Service d′Épidémiologie, CHU de Sétif, 1900 Sétif, Algeria; 8 Service de Radiothérapie, Institut National d′Oncologie, BP 3213, Rabat, Morocco; 9 Département de Chimie et Sciences de la Vie, Université Saint-Esprit de Kaslik Faculté des Sciences et de Génie Informatique, BP 446, Jounieh, Lebanon; 10 Laboratoire d′Immuno-oncologie Moléculaire, Faculté de Médecine de Monastir, 5019 Monastir, Tunisia; 11 Service de Radiothérapie, Centre d′Oncologie IBN Rochd, Casablanca, Morocco; 12 International Network for Cancer Treatment and Research, B-1180 Brussels, Belgium

**Keywords:** North Africa, nasopharyngeal carcinoma, tobacco, cannabis, fumes

## Abstract

**Background:**

The lifestyle risk factors for nasopharyngeal carcinoma (NPC) in North Africa are not known.

**Methods:**

From 2002 to 2005, we interviewed 636 patients and 615 controls from Algeria, Morocco and Tunisia, frequency-matched by centre, age, sex, and childhood household type (urban/rural). Conditional logistic regression was used to evaluate the association of lifestyles with NPC risk, controlling for socioeconomic status and dietary risk factors.

**Results:**

Cigarette smoking and snuff (tobacco powder with additives) intake were significantly associated with differentiated NPC but not with undifferentiated carcinoma (UCNT), which is the major histological type of NPC in these populations. As demonstrated by a stratified permutation test and by conditional logistic regression, marijuana smoking significantly elevated NPC risk independently of cigarette smoking, suggesting dissimilar carcinogenic mechanisms between cannabis and tobacco. Domestic cooking fumes intake by using *kanoun* (compact charcoal oven) during childhood increased NPC risk, whereas exposure during adulthood had less effect. Neither alcohol nor *shisha* (water pipe) was associated with risk.

**Conclusion:**

Tobacco, cannabis and domestic cooking fumes intake are risk factors for NPC in western North Africa.

Nasopharyngeal carcinoma (NPC) is a rare malignancy in most regions of the world, but is significantly more common in parts of Southeast Asia, western North Africa (Algeria, Morocco and Tunisia) and within the artic circle of North America. Major risk factors include Epstein–Barr virus (EBV) infection, genetic susceptibility, diet and other environmental exposures (Hildesheim and Levine, 1993; Jeannel *et al*, 1999; Yu and Yuan, 2002; Busson *et al*, 2004; Chang and Adami, 2006). In North Africa, cigarette smoking, alcohol consumption and cooking in the main living room during childhood have been suggested to be associated with increased NPC risk (Jeannel *et al*, 1990; Ammor *et al*, 2003). However, these studies are far from conclusive because of inadequate adjustment for potential confounding factors, small sample size (⩽80 cases) and the findings not being significant.

Recently, we carried out a large multi-centric case–control study in Algeria, Morocco and Tunisia of genetic and environmental risk factors for NPC (Feng *et al*, 2007). In this study, we examined NPC risk in relation to tobacco, cannabis, domestic fumes and alcohol intake, taking into account the effects of associated dietary risk factors, socioeconomic status (SES) and other potential confounders; lifestyles specific to North Africa, such as local cooking practice facilities and local forms of tobacco intake were investigated in detail.

## Materials and methods

Details of the studied populations are described elsewhere (Feng *et al*, 2007). In brief, all incident cases diagnosed in 2001–2004 in five hospitals were identified by clinicians in the oncology and radiotherapy departments, and were invited for interview. In addition, some prevalent cases were also recruited, comprising 25% of the total. Controls were hospitalised individuals from 15 non-cancer hospital departments (61%) or friends and family members of non-NPC cancer patients (39%), frequency-matched by centre, age, sex, and childhood household type (urban/rural). Hospitalised controls were recruited from selected departments (see Feng *et al*, 2007), in which diseases sharing risk factors with NPC were carefully excluded (i.e., ear, nose, and throat conditions; alcohol- and tobacco-related diseases; or potential HLA-related disorders such as autoimmune diseases). Among the individuals invited to participate (both cases and controls), more than 90% were successfully interviewed. The primary reason for non-participation was old age. Informed consent was obtained from each participant, and the International Agency for Research on Cancer ethical committee approved the study protocol.

In each centre, trained doctors conducted the interviews using identical questionnaires; histological type and other details were collected from medical records. Questions with regard to cigarette smoking included age at initiation, age at successful quitting, total time of cessation in between, and number of cigarettes smoked per day. Other forms of tobacco intake, such as pipe, *shisha* (*hookah*, water pipe) and *neffa* (snuff, a powdered mix of tobacco leaf, calcium phosphate and lime, consumed by sniffing or chewing) were assessed separately from cigarette smoking. Alcohol consumption information included age at initiation and cups per week for five categories: beer/cider, wine, aperitifs, liqueurs, and whisky/digestive. Average alcohol percentages by volume in these beverages were estimated as 6.5, 12.5, 20, 32, and 45%, and the average cup volume was 25cl, 12cl, 4cl, 4cl and 4cl, separately. The total ethanol intake in 1 week was then calculated. Questions regarding cannabis consumption included its form (herbal, resinous, oily, or other), mode of consumption (smoked, smoked with tobacco, ingested, drunk, sniffed, or inhaled), frequency (times per month), and age at initiation and at quitting. Interviewers, all among the present researchers, were trained to be sensitive to the stigma attached to cannabis and tobacco consumption that exists within their countries, and were highly committed to obtaining accurate answers.

Sources of fumes intake in North Africa are diverse, and include burnt perfume, cooking with *kanoun* (a light and compact-sized oven that runs on charcoal) or *tabouna* (a traditional oven in North Africa and Middle East), wood fire cooking, wood fire heating, or occupational fumes intake. Our questionnaire data included these exposures during childhood and adulthood, and the kitchen ventilation status that may affect fumes intake; the latter was classified as ventilated (presence of windows or chimney in the kitchen, or cooking in open air) or unventilated (absence of windows or chimney in the kitchen, or cooking in the main living space).

### Statistical analyses

All statistical analyses were carried out using STATA 9.0 (STATA Corp. College Station, TX, USA). Conditional logistic regression with strata defined by sex and centre was used to evaluate the association of a specific factor with NPC risk, adjusting for age, SES variables (household type, lodging category, occupation and education level) and associated dietary factors (rancid butter, rancid sheep fat and cooked vegetables during adulthood) (Feng *et al*, 2007). Odds ratios (OR) and corresponding 95% confidence intervals (CI) were calculated. For variables with more than two levels of ordered exposures, the Armitage trend test was used to assess significance. Considering that in these countries tobacco consumption is generally under-reported in women who are also exposed to passive tobacco smoking at home, analyses on tobacco consumption were restricted to men. Similarly, only males were considered in analyses of marijuana smoking, using a stratified permutation test to control for the strong confounding effect of cigarette smoking in addition to SES and diet (Manichaikul *et al*, 2007), which was performed as follows: first, the OR for cannabis consumption (denoted as OR^*^) was calculated by conditional logistic regression adjusting for age, SES, and diet; next, the variable for cannabis exposure was permuted in the smoker and non-smoker strata independently, so that the correlation between cannabis and cigarette smoking status is reserved. The OR for this permuted variable was then calculated in the same way as that for OR^*^. Finally, from 10 000 repeats of the previous step, one-sided *P*-value was calculated as the proportion of sampled permutations in which OR is equal to or higher than OR^*^. Simulation has shown that this method has a correct type I error rate and is more powerful in association detection, controlling for a strong confounder than logistic regression adjusting for the confounder (data not shown). A male-only multivariate analysis was performed by stepwise inclusion, adjusting for age and SES, starting with associated dietary risk factors and significant variables such as tobacco, alcohol, cannabis, and domestic fumes exposure.

## Results

From January 2002 to March 2005, a total of 636 cases and 615 controls were interviewed. Details of demographic breakdown by sex, age, and study centre were presented earlier (Feng *et al*, 2007). In summary, there were no significant differences between cases and controls with regard to ethnic group, country, recruitment centre, or household type during childhood. Almost all (587, 92%) cases were non-keratinising undifferentiated carcinomas (UCNT), the rest were 14 (2.2%) non-keratinising differentiated carcinomas, 5 (0.79%) keratinising squamous cell carcinomas, and 30 (4.7%) of unknown histological type.

Table 1 presents the association of NPC risk with cigarette smoking and snuff intake in men. After adjustment for SES and dietary risk factors, the OR of ever smoking compared with non-smoking was not significant at 1.3. Age at initiation and quitting, and aggregate smoking history were not significantly associated; however, dose of cigarette intake (numbers per day) was significantly associated with increased NPC risk by trend test (*P*<0.05). When analyses were restricted to UCNT, results were similar, but no variable remained significant, with the trend *P*-value for cigarettes smoked per day being 0.10. On the contrary, despite the much smaller numbers (14 patients), dose of cigarette intake was significantly associated with differentiated NPC (*P*<0.05; Table 1).

More snuff was consumed in rural areas than in cities (*P*<0.0001), in populations with less education (*P*<0.0001), lower occupation level (*P*<0.002), or smaller lodging (*P*<0.004), pointing to an association with lower SES. Among snuff consumers, 70% chewed and 31% sniffed, with very few engaging in both (1.4%). After adjustment for SES and diet, association of snuff with NPC risk was far from significant, and so was the dose response, as were analyses restricted to UCNT. However, similar to cigarette smoking, snuff intake was significantly associated with differentiated NPC, although the numbers were small; the trend of risk with a higher dose of snuff intake was, however, not significant (*P*=0.20; Table 1).

In contrast to snuff, *shisha* was associated with higher SES, such as higher education (*P*<0.0005), higher occupation level (*P*<0.01), better living conditions (*P*<0.03) and urban household type (*P*<0.05). *Shisha* showed no association with NPC or UCNT, whereas with differentiated NPC, this could not be tested because of small numbers (Table 1).

Cannabis was more often consumed by individuals with smaller lodging (*P*<0.002), less education (*P*<0.009) or lower occupation level (*P*<0.009), suggesting an association with lower SES. Almost all cannabis users also smoked cigarettes (97%), but among the individuals who consumed both, the dose correlation between cigarette smoking and cannabis was not significant (Spearman's rho= −0.13, *P*>0.05). After adjustment for age, SES and diet, cannabis intake was significantly associated with higher NPC risk and with a significant dose–response relationship (Table 2). Results were similar for UCNT, but were not significant for differentiated NPC (results not shown). After adjusting for the number of cigarettes smoked per day, association was significant for high-dose lifetime consumption (at least 2000 times), but cannabis intake as an ever/never variable was no longer significantly associated (Table 2). However, using stratified permutation, a more powerful method to account for the confounding by cigarette smoking, ever consumption of cannabis was significantly associated with increased NPC risk (*P*<0.025).

There was no association of NPC with alcohol consumption assessed as ever/never, either in crude analyses or after adjusting for SES and diet (OR=1.2 (0.8–1.6)). There was no evidence of a dose response when specific quantities were examined, nor was there an association by type of alcohol consumed (*P*=0.60 for beer; *P*=0.90 for all alcoholic beverages) or by histological type of NPC.

Occupational fumes intake was not common among the studied population, only 35 cases (6%) and 16 controls (2%) responded that they were exposed to fumes as a part of their employment, and no significant effect was found (OR=1.5 (0.8–2.9)). However, domestic fumes exposure from usage of a *kanoun* oven during childhood was associated with increased NPC risk, remaining significant after adjustment for SES and associated dietary elements (OR=1.86 (1.28–2.72); Table 3). In addition, higher fumes exposure by cooking with wood fire or cooking in a poorly ventilated room during childhood was more frequent in cases than in controls, although not significantly (Table 3). Restricting the cases to UCNT patients did not appreciably affect the findings.

In our previous dietary investigation, we showed that consumption of rancid butter and rancid sheep fat was associated with increased NPC risk, whereas that of cooked vegetables was protective (Feng *et al*, 2007). In a male-only multivariate analysis covering these dietary factors, adjusting for age and SES, with an inclusion criteria of *P*<0.05, the variables remaining in the final model indicated an independent contribution of consumption of rancid butter, rancid sheep fat and cooked vegetables during adulthood, kitchen ventilation during childhood and lifetime cannabis consumption to NPC risk (Table 4).

## Discussion

In our analyses, SES could be a strong confounder in all studied lifestyle factors. SES confounding effects were controlled by matching controls with cases according to rural/urban household, and adjusting for the six SES variables, as previously described in detail (Feng *et al*, 2007). Potential bias due to the inclusion of prevalent cases and hospitalised controls was expected to be minor (Feng *et al*, 2007). Another source of potential confounding was diet. Many studies have reported that smokers tend to eat more saturated fat and less fresh fruit or vegetables than do non-smokers (Dallongeville *et al*, 1998). As alcohol consumption and cooking methods could also be related to diet, we adjusted for the significantly associated foods from our previous multivariate analyses to minimise confounding, while adjusting for as few variables as possible.

The studied countries prohibit cannabis consumption, hence the reported consumption was possibly underestimated. Nevertheless, as there is no perceived relationship between cannabis and cancer in the area, under-reporting of cannabis consumption was presumably similar in cases and in controls, which could only decrease the significance of the association rather than create a spurious one. Finally, as in any retrospective study, our findings are subject to recall bias. However, this problem should be limited to those factors involving tobacco, as the studied populations were not familiar with possible links between cancer and alcohol, cannabis or domestic fumes intake.

Despite a small sample size, cigarette smoking was significantly associated with differentiated NPC, which is in agreement with the findings from North America (Mabuchi *et al*, 1985; Nam *et al*, 1992; Chow *et al*, 1993; Vaughan *et al*, 1996; Zhu *et al*, 1997), where differentiated NPC predominates (Ou *et al*, 2007). Although the association with UCNT was not significant, a trend of increased risk of UCNT with increased dose of cigarette intake per day was apparent, leaving the question unresolved. Case–control studies from endemic areas (mainly from Asia), where the vast majority of NPCs are UCNTs, have produced conflicting results (Chen *et al*, 1990; Ning *et al*, 1990; Yu *et al*, 1990; Cheng *et al*, 1999; Armstrong *et al*, 2000; Yuan *et al*, 2000; Zou *et al*, 2000; Friborg *et al*, 2007; Guo *et al*, 2009). Our findings point to the importance of separating UCNT and non-UCNT in analyses. However, the lack of well-trained histologists in many endemic areas may result in some non-UCNTs being mistakenly categorised as UCNTs, and our study may not be free of such bias.

Similar to cigarette smoking, snuff was significantly associated with differentiated NPC, but not with UCNT. This is noteworthy, as it contrasts with the locally held view that snuff is an important risk factor for NPC because of its tobacco nature and its direct contact with the nasopharynx. Our results represent further evidence of the harmful effect of tobacco consumption on differentiated NPC; but as this constitutes a small proportion of all NPCs in North Africa, the contribution of cigarette and snuff to general NPC risk in these countries is small.

Epidemiological studies on cannabis have been difficult, mainly because of the strong confounding effect of cigarette smoking. In fact, most studies did not yield significant results after adjustment for tobacco use (Hashibe *et al*, 2005), as also in our study. However, a stratified permutation analysis showed that, independent of cigarette smoking, cannabis significantly elevates NPC risk. In contrast to cigarette or snuff intake, cannabis consumption was significantly associated with UCNT but not with differentiated NPC, suggesting that the carcinogenic mechanisms of cannabis may differ from those of tobacco.

Our results indicate that usage of traditional cooking facilities (*kanoun*) during childhood increases NPC risk almost two-fold, whereas during adulthood, it seems to have less effect. This difference cannot be explained by the aggregate exposure time, because the NPC risk remained significantly elevated among individuals who used *kanoun* during childhood but not during adulthood (OR=1.80 (1.21–2.68)). Poor ventilation of the kitchen and wood fire cooking during childhood were associated with a 30–40% increased risk of NPC, although these did not reach statistical significance. Moreover, during childhood, the excess risk associated with *kanoun* cooking increased when the kitchen was not ventilated (OR=2.44 (1.47–4.05)). These results, further supported by a multivariate analysis, suggest that domestic fumes exposure during childhood is a risk factor for NPC in North Africa. They corroborate the positive associations reported in endemic populations of Asia (Shanmugaratnam *et al*, 1978; Zheng *et al*, 1994; Guo *et al*, 2009).

## Conclusions

This, the first large-scale case–control study in the North African endemic area of NPC, shows evidence that NPC risk is increased by tobacco consumption, marijuana smoking and domestic cooking fumes intake.

## Figures and Tables

**Table 1:** Association of cigarette smoking, snuff intake, and *shisha* consumption with NPC risk in men

**Exposure**	**Controls**	**All patients**			**UCNTs**			**Differentiated NPCs**		
**Levels**	***N* (%)**	***N* (%)**	**OR (95%CI)**	**Trend-*P***	***N* (%)**	**OR (95%CI)**	**Trend-*P***	***N* (%)**	**OR (95%CI)**	**Trend-*P***
*Cigarette smoking*										
Never	174 (43)	161 (37)	1		152 (37)	1		5 (36)	1	
Ever	235 (57)	279 (63)	1.28 (0.93–1.77)		254 (63)	1.21 (0.87–1.68)		9 (64)	3.3 (0.30–35.8)	
*Smoking history*				0.25			0.36			0.29
Never	174 (43)	161 (37)	1		152 (37)	1		5 (36)	1	
1–20 years	106 (26)	125 (28)	1.40 (0.94–2.09)		111 (27)	1.28 (0.85–1.93)		5 (36)	2.37 (0.13–44.6)	
>20 years	121 (30)	154 (35)	1.22 (0.84–1.79)		143 (35)	1.18 (0.80–1.73)		4 (29)	3.80 (0.32–45.6)	
*Cigarettes per day*				**0.05**			0.10			**0.05**
None	174 (43)	161 (37)	1		152 (37)	1		5 (36)	1	
1–12	80 (20)	81 (18)	1.09 (0.70–1.68)		72 (18)	1.01 (0.65–1.59)		3 (21)	0.58 (0.00–105)	
13–22	117 (29)	135 (31)	1.30 (0.89–1.90)		124 (31)	1.24 (0.84–1.82)		3 (21)	0.13 (0.00–11.6)	
>22	38 (9)	63 (14)	1.62 (0.97–2.72)		58 (14)	1.52 (0.89–2.58)		3 (21)	**313** (**1.94–50336**)	
										
*Snuff intake*										
Never	350 (84)	378 (84)	1		352 (85)	1		8 (57)	1	
Ever	69 (16)	71 (16)	1.03 (0.64–1.65)		63 (15)	0.97 (0.59–1.58)		6 (43)	**30.2** (**1.67–546**)	
*Snuff bags per week*				0.99			0.97			0.20
None	350 (84)	378 (86)	1		352 (86)	1		8 (62)	1	
1	26 (6)	17 (4)	0.67 (0.33–1.38)		15 (4)	0.64 (0.31–1.34)		2 (15)	5.19 (0.07–360)	
>1	40 (10)	45 (10)	1.08 (0.60–1.94)		41 (10)	1.07 (0.58–1.98)		3 (23)	10.2 (0.26–400)	
										
*Shisha* consumption										
Never	404 (96)	441 (98)	1		407 (98)	1		14 (100)	1	
Ever	15 (4)	9 (2)	0.49 (0.20–1.23)		9 (2)	0.50 (0.20–1.24)		0 (0)	–	

Abbreviations: CI=confidence interval; NPC=nasopharyngeal carcinoma; OR=odds ratio; UCNT=undifferentiated carcinoma of nasopharyngeal type.

Note: Analyses were stratified by sex and centre, adjusted for age, SES and associated dietary factors. Significant test results are shown in bold fonts.

**Table 2:** Association of cannabis consumption with NPC risk in men

	**Controls**	**Cases**	**Without adjustment for cigarette smoking** a		**Adjusted for cigarette smoking** b	
**Exposure levels**	***N* (%)**	***N* (%)**	**OR (95%CI)**	**Trend-*P***	**OR (95%CI)**	**Trend-*P***
*Cannabis consumption*						
Never (ref.)	385 (94)	387 (88)	1		1	
Ever	26 (6)	52 (12)	**1.83** (**1.04–3.22**)		1.54 (0.86–2.76)	
*Frequency of consumption*				**0.04**		0.10
Never (ref.)	385 (94)	387 (90)	1		1	
<30 times per month	9 (2)	17 (4)	1.65 (0.69–3.91)		1.40 (0.58–3.36)	
⩾30 times per month	14 (3)	27(6)	2.09 (0.98–4.50)		1.85 (0.86–4.02)	
*Lifetime consumption*				0.007		0.02
Never (ref.)	385 (96)	387 (90)	1		1	
<2000 times	10 (2)	20 (5)	2.14 (0.92–4.98)		1.86 (0.79–4.36)	
⩾2000 times	7 (2)	21 (5)	**2.97** (**1.15–7.71**)		**2.62** (**1.00–6.86**)	
						
*Mode of consumption* c						
Never (ref.)	385 (94)	387 (89)	1		1	
Smoking	9 (2)	12 (3)	1.11 (0.43–2.88)		0.97 (0.37–2.52)	
Smoking with Tobacco (*kif*)	15 (4)	38 (9)	**2.33** (**1.17–4.63**)		1.94 (0.96–3.92)	

Abbreviations: CI=confidence interval; NPC=nasopharyngeal carcinoma; OR=odds ratio.

Note: Significant test results are shown in bold fonts.

a Analyses were stratified by sex and centre, adjusted for age, SES measures, and associated dietary factors.

b Analyses were stratified by sex and centre, adjusted for age, SES measures, associated dietary factors, and cigarettes smoked per day.

c Ingestion and other mode of consumption were not included because of the low frequency of observations.

**Table 3:** Association of domestic fumes intake with NPC risk in men and women

	**Childhood**			**Adulthood**		
**Exposure levels**	**Control (%)**	**Case (%)**	**OR** (**95%CI)**	**Control (%)**	**Case (%)**	**OR** (**95%CI)**
*Kanoun*						
Never (ref.)	203 (33)	140 (22)	1	365 (65)	319 (56)	1
Ever	408 (67)	485 (78)	**1.86** (**1.28–2.72**)	197 (35)	251 (44)	1.41 (0.98–2.04)
						
*Tabouna*						
Never (ref.)	273 (46)	263 (43)	1	362 (66)	370 (65)	1
Ever	318 (54)	355 (57)	1.12 (0.81–1.54)	188 (34)	199 (35)	1.01 (0.72–1.43)
						
*Wood fire cooking*						
Never (ref.)	257 (43)	217 (37)	1	455 (83)	423 (80)	1
Ever	335 (57)	368 (63)	1.38 (0.95–2.00)	93 (17)	108 (20)	1.23 (0.82–1.85)
						
*Wood fire heating*						
Never (ref.)	272 (46)	251 (44)	1	450 (82)	412 (78)	1
Ever	321 (54)	324 (56)	1.01 (0.71–1.42)	98 (18)	113 (22)	1.17 (0.79–1.72)
						
*Kitchen ventilation* a						
Ventilated (ref.)	495 (81)	469 (75)	1	492 (88)	507 (90)	1
Not ventilated	113 (19)	154 (25)	1.29 (0.93–1.80)	65 (12)	58 (10)	0.78 (0.51–1.20)

Abbreviations: CI=confidence interval; NPC=nasopharyngeal carcinoma; OR=odds ratio.

Note: Analyses were stratified by sex and centre, adjusted for age, SES measures, and associated dietary factors. Significant test results are shown in bold fonts.

a ‘Ventilated’ means chimney or windows present in kitchen, or cooking in open air; ‘Not ventilated’ means chimney or windows absent in kitchen, or cooking in the main living room.

**Table 4:** Associated risk factors in a multivariate model in men

**Exposures**	**OR (95%CI)**	***P*-value**
*Rancid butter (Adulthood)*		0.001
<10 times per year (ref.)	1	
<3 times per week	1.44 (0.93–2.23)	
⩾3 times per week	3.52 (1.71–7.23)	
		
Rancid sheep fat (Adulthood)		0.004
<10 times per year (ref.)	1	
<3 times per week	2.08 (1.33–3.26)	
⩾3 times per week	1.70 (0.85–7.23)	
		
*Cooked vegetables (Adulthood)*		0.004
<3 times per week (ref.)	1	
⩾3 times per week	0.54 (0.36–0.82)	
		
*Kitchen ventilation (Childhood)*		0.02
Ventilated (ref.)	1	
Not ventilated	1.64 (1.09–2.48)	
		
*Lifetime cannabis consumption*		0.007
Never (ref.)	1	
<2000 times	2.00 (0.85–4.70)	
⩾2000 times	3.25 (1.24–8.50)	

Abbreviations: CI=confidence interval; NPC=nasopharyngeal carcinoma; OR=odds ratio.

Note: Analyses were stratified by sex and centre, adjusted for age and SES, with female samples excluded. Stepwise inclusion method was used, with inclusion criteria of *P*<0.05. When there are more than two exposure levels, *P*-values correspond to trend test.
